# Causes and Consequences of Chromatin Variation between Inbred Mice

**DOI:** 10.1371/journal.pgen.1003570

**Published:** 2013-06-13

**Authors:** Mona Hosseini, Leo Goodstadt, Jim R. Hughes, Monika S. Kowalczyk, Marco de Gobbi, Georg W. Otto, Richard R. Copley, Richard Mott, Douglas R. Higgs, Jonathan Flint

**Affiliations:** 1 Wellcome Trust Centre for Human Genetics, Oxford, United Kingdom; 2 MRC Molecular Haematology Unit, Weatherall Institute of Molecular Medicine, University of Oxford, Oxford, United Kingdom; University of North Carolina, United States of America

## Abstract

Variation at regulatory elements, identified through hypersensitivity to digestion by DNase I, is believed to contribute to variation in complex traits, but the extent and consequences of this variation are poorly characterized. Analysis of terminally differentiated erythroblasts in eight inbred strains of mice identified reproducible variation at approximately 6% of DNase I hypersensitive sites (DHS). Only 30% of such variable DHS contain a sequence variant predictive of site variation. Nevertheless, sequence variants within variable DHS are more likely to be associated with complex traits than those in non-variant DHS, and variants associated with complex traits preferentially occur in variable DHS. Changes at a small proportion (less than 10%) of variable DHS are associated with changes in nearby transcriptional activity. Our results show that whilst DNA sequence variation is not the major determinant of variation in open chromatin, where such variants exist they are likely to be causal for complex traits.

## Author Summary

Regulatory sites of the genome affect gene expression and complex traits, including disease susceptibility. Variable regulatory sites are potentially interesting because they are a likely cause of phenotypic variation, providing a bridge between sequence and transcriptional variation. In this paper we identify regions of the genome where DNA is not wrapped up in chromatin (hence potentially regulatory) in eight inbred strains of mice. We compare sites that vary among strains and compare them to non-variable sites. We show that more than half of variable sites cannot be attributed to local sequence variation. Functional consequences (in terms of readily detectable changes in gene expression) are associated with less than 10% of variable DNase I hypersensitive sites. We show that variable sites are enriched for sequence variants contributing to complex traits in mice.

## Introduction

Deoxyribonuclease I (DNase I) hypersensitive sites (DHS) mark alterations in chromatin structure associated with active regions of regulatory DNA [1]. Tissues differ in the genomic distribution of DHS, reflecting variation in tissue specific regulatory factors [2]–[4], but less is known about the extent and causes of variation between individuals. In this paper we address the relationship between sequence variation, DHS variation and phenotypic variation.

Two previous studies have estimated individual variation in regulatory regions in human lymphoblastoid cell lines: 10% of DHS displayed inter-individual variation [5] and 25% of binding sites for RNA polymerase II (sites that will appear in surveys of DHS) [6]. Both studies identified underlying sequence variation as an important contributor, but could not determine whether sequence variation was the major contributor. For example, the total fraction of significant binding differences coinciding with genetic variations was 26% for RNA polymerase II [6], leaving the possibility open that non-genetic causes are an important cause of variation at regulatory sites.

Variation in regulatory elements is thought to have functional consequences: thus sequence variation could give rise to new functional elements, which in turn would alter gene expression and result in phenotypic variation. In one example a single nucleotide polymorphism (SNP) upstream of the human α-globin genes created a new promoter-like element between the globin gene upstream regulatory elements and their cognate promoters. This element caused significant down-regulation of the α-globin genes, resulting in an inherited anemia [7], demonstrating the potential importance of the mechanism, but leaving open the question of its prevalence. It is possible that many variable DHS are without readily detectable phenotypic consequences.

A recent genome-wide survey of DHS in lymphoblastoid cell lines confirmed the importance of sequence variation as a source of DHS variation, and reported that 16% of DHS associated with local sequence variants were also associated with variation in transcript abundance at neighbouring genes [8]. Potentially therefore these 16% might contribute to phenotypic variation. Many of the genetic signals identified through genome wide association studies (GWAS) for complex traits and disease susceptibility reside within DHS [2], [9], but whether these signals preferentially exist at variable DHS is not known.

We set out to estimate the proportion of heritable variation in DHS, to estimate how much of this is captured by local sequence variation, and assess the consequences of variation on both gene expression and phenotypic variation. To do so, we chose a relatively homogeneous, primary tissue type (terminally differentiated erythroblasts) obtained from eight inbred mouse strains (A/J, AKR/J, BALB/cJ, C3H/HeJ, C57BL/6J, CBA/J, DBA/2J and LP/J) for which availability of near complete sequence of all eight strains permitted identification of virtually all sequence variants contributing to DHS variation [10], [11]. Furthermore, 843 quantitative trait loci (QTLs) have been identified in over 2,000 heterogeneous stock (HS) mice descended from these strains [12]. The traits mapped included disease models (asthma, anxiety and type 2 diabetes), as well as haematological, immunological, biochemical and anatomical assays. We were thus able to ask whether sequence variants contributing to DHS variation are also likely to influence quantitative traits [13].

## Results

**Figure 1:**
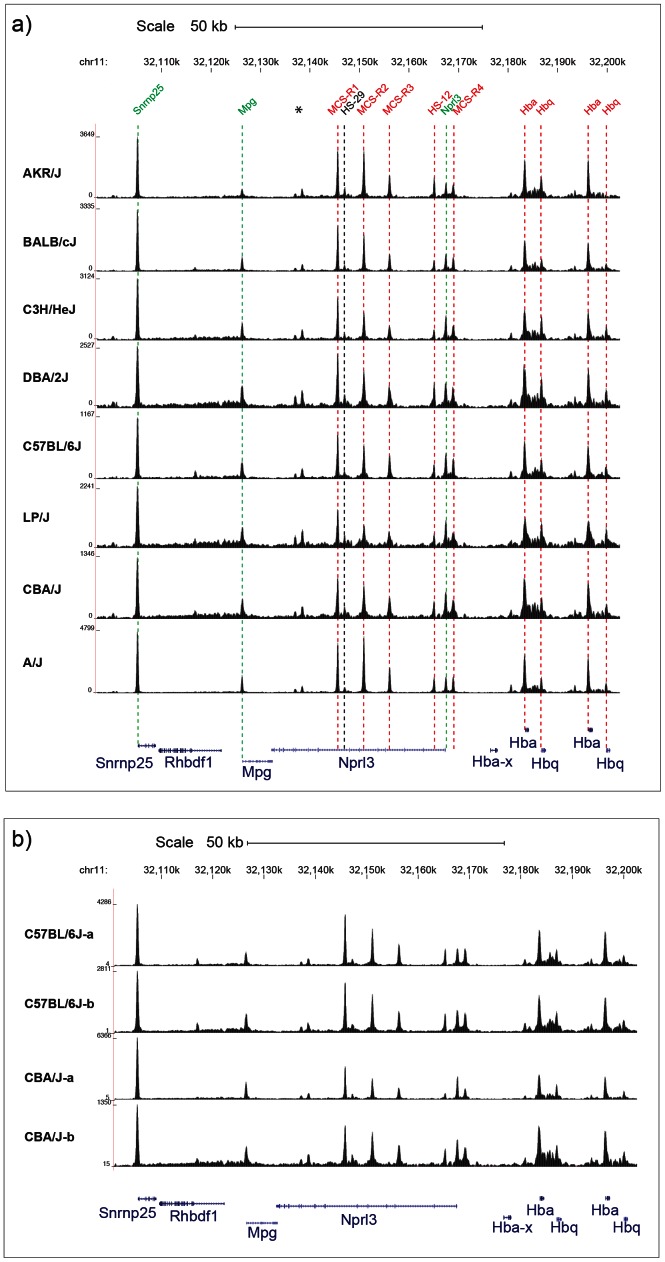
DNase I Hypersensitive sites at the α globin cluster on chromosome 11. **a**) Chromosomal positions in Kb on chromosome 11 are shown for MGSC37 mouse reference genome. The names of previously annotated *DNaseI* hypersensitive sites (DHS) are shown coloured as ubiquitous (green), erythroid (red) and black (CTCF) [15]. Two previously unpublished DHS (marked *) are associated with CTCF binding sites. The density of aligned DHS-seq reads in a moving 300 bp window, with a 30 bp increment is shown for each mouse strain. DHS-seq peaks are aligned with the position of each previously described DHS by dashed lines, colour coded as described. The position of Refseq annotated genes are named and shown below in blue. **b**) Two examples of DHS-seq biological replicates for the C57BL/6J and CBA/J strains are shown over the same region as in Figure 1a.

We generated DHS data using 19 mice from eight inbred strains (three each of C57BL/6J, A/J, and CBA/J mice, and two each of AKR/J, C3H/HeJ, DBA/2, BALBc/J, and LP/J strains). We avoided alignment biases towards alleles present in the reference genome (C57BL/6J) by creating strain specific reference sequences using known SNPs [10], [11]. Figure 1a shows normalized counts of aligned reads for the eight inbred strains over a 100 Kb region containing the previously well characterized alpha globin gene cluster on chromosome 11 [14]. DHS for each strain are identical and agree on the location of previously mapped DHS (the position of known DHS are shown by vertical lines) [15]. Furthermore within-strain DHS, mapped in two entirely independent experiments, are highly concordant (Figure 1b). After adjusting for local variation in the coverage of aligned reads, we called DHS by searching for regions with high numbers of aligned reads (hereafter peak heights) [16]. Across all 19 experiments there was a total of 36,693 DHS that covered 9.1 Mb (0.29% of the genome); 25,700 peaks (6.3 Mb) lay within mappable (single copy) regions of the genome and we used this subset for further analyses.

We carried out a genome-wide quantification of heritable peak variation by comparing variation in peak height between and within strains to find peaks where the pattern of variation between strains was consistent across biological replicates. Variation between peaks was estimated under the assumption that the distribution of reads at a peak can be modeled as a negative binomial distribution, using DESeq software [17]. At a false discovery rate (FDR) of 10% we found 2,530 variable peaks (9.8%), and 1,397 peaks at a 1% FDR (5.4%). We checked the automated detection of variable peaks by visual inspection of peaks on chromosomes 14 to 19 and confirmed all were detected in the automated analysis (Table S1). We found that across a wide range of parameter values the proportion of variable peaks detected automatically remained approximately constant (Figure S1). We refer to these 1,397 DHS as the set of variable DHS (Table S1).

**Figure 2:**
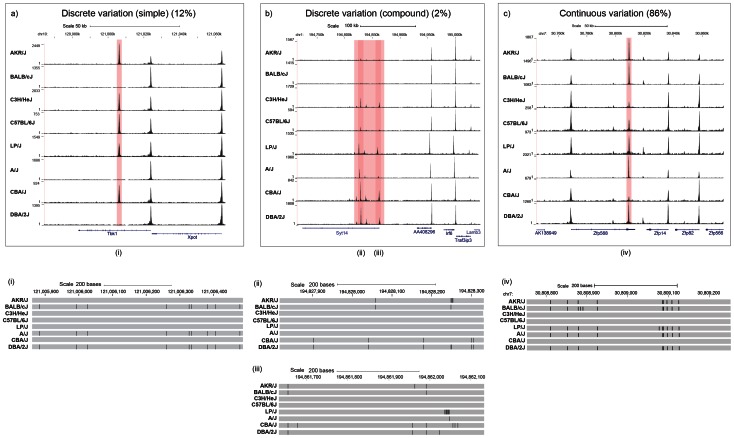
Three categories of variable DNase I hypersensitive sites. (**a**) Discrete variation (simple): DHS entirely absent in one or more strains. The position of a DHS missing in one or more strains is highlighted in red. The position of annotated Refseq genes are named and shown in blue. The strain specific genotype of the DNA underlying the variable peaks is shown below with a grey bar representing the portions identical to the current mm9 build; differences are represented as black lines. **b**) Discrete variation (compound): clusters of variable DHS. Positions of DHS that vary together with the underlying strain differences are displayed as described for panel a). **c**) Continuous variation: DHS vary in shape or size. The position of DHS sites together with underlying strain differences are displayed as described for panel a). The top part of each panel shows chromosomal positions in kilobases (Kb) (MGSC37 mouse reference), while the lower section gives the chromosomal positions in base pairs (bp) over the highlighted DHS. The density of aligned DHS-seq reads in a moving 300 bp window, with a 30 bp increment is shown for each mouse strain.

Visual inspection of the variable peaks revealed that the majority (86%) differ in peak height and shape (continuous variation). A minority of variable peaks (196 peaks, 14%) display discrete variation between strains, with a loss (or gain) of a peak in one or more strains. A proportion of the latter were found to occur in clusters, characterized by a number of closely linked peaks (defined here as within 10 Kb) present in one strain, but absent in others. We identified 23 of these discrete compound variable peaks (1.6% of the total) and 173 discrete unique variable peaks (12.3% of the total). Examples are given in Figure 2.

We estimated how much of the variation might be attributable to underlying sequence differences by calculating heritability from between and within strain variation [18]. Heritability was defined as half the between strain component of variance divided by the sum of the within strains variance and half the between strain variance. The mean heritability of the variable peaks is 69.4%.

We compared the 1,397 variable DHS to 2,849 with least evidence for variation (P-value >0.97) and noted the following features of variable DHS (Table 1). First, they contain more sequence variants: 75% contain one or more previously identified sequence variants within 100 bp of the edges of the peak compared to 58.3% of non-variable DHS (P-value = 4.3e-27, Fisher exact test). For this and subsequent analysis of sequence variants we analysed all classes of variation, SNPs, insertion deletion polymorphisms and structural variants [10], [11]. Second, variable DHS are less likely to coincide with conserved regions (using the multiple alignment of 30 vertebrate species and measurements of evolutionary conservation, UCSC browser track) of the mouse genome (61.5% compared to 71.4%, P-value = 1.07e-10 (Fisher exact test)). Third, variable DHS were more likely to be associated with chromatin that has the signature of an enhancer (20.7% compared to 9.7%, P-value = 5.6e-22 (Fisher exact test)), rather than a promoter (25.5% compared to 52.2%, P-value = 3.1e-63 (Fisher exact test)). Here enhancers were classified as DHS overlapping a genomic region enriched for H3K4me1 and promoters for H3K4me3 enrichment in terminally differentiated mouse erythroblasts, using data from [15].

**Table 1:** Characteristics of variable Deoxyribonuclease I hypersensitive sites.

Peak type	Number of peaks	Variants (%)	LogP	Conservation (%)	LogP	Enhancer (%)	LogP	Promoter (%)	LogP
Most variable peaks (P value <0.0008)	1,396	75.00		61.53		20.63		25.50	
All peaks (P value >0.008)	24,303	61.05	25.02	70.04	10.04	13.91	10.13	46.22	52.01
All peaks (P value >0.5)	16,299	59.11	28.95	71.37	12.09	10.78	20.85	50.74	68.33
Least variable peaks (P value >0.97)	2,849	58.25	26.37	71.43	9.98	9.72	20.84	52.12	62.07

The table shows features of the most variable Deoxyribonuclease I hypersensitive sites (identified at FDR threshold of 1%, equivalent to an adjusted P-value of 0.008), compared to non variable peaks. The features shown are the percentage of sites containing sequence variants (Variants), the percentage of sites showing sequence conservation (Conservation), the percentage of sites with the signature of an enhancer (Enhancer) and those with the signature of a promoter (Promoter). The P-value is that obtained from estimating peak differences between strains (modeled as a negative binomial distribution). Results are shown for comparisons between the most variable peaks and three sets of non-variable peaks, defined at different stringencies (P-values>0.008, 0.5 and 0.97). The significance of the comparison is shown as the negative logarithm (base 10) of the P-value of a Fisher exact test.

It is possible that our comparison between variable and non-variable peaks is biased by the choice of an extremely non-variable set of peaks. Therefore we compared variable peaks with all other peaks (excluding the most variable) and with the 16,299 peaks where analysis of variation gave a P-value of 0.5 or greater. The results, presented in Table 1 show similarly highly significant differences in the same features as those obtained from the most non-variable sites. Note that as the set of sites becomes less variable (as indicated by an increasing P-value in column one of the table) the relative enrichment in promoter elements and conservation increases, as does the relative impoverishment in enhancers and sequence variant. This is consistent with the hypothesis that the 2,849 DHS with least evidence for variation (P-value >0.97) are simply an extreme, rather than an atypical set.

To identify the probable causal variants for DHS variation, we correlated sequence and site variation. Causal variants will be those with the same strain distribution pattern (SDP) as the DHS (i.e. strains with similar peak heights must have the same causal allele). This method of haplotype association mapping is appropriate when searching for a local effect (in-*cis*), but will not allow us to detect *trans* effects. An example is shown in Figure 2a. However, it should be noted that large shared haplotype blocks in the inbred strains used here typically make it impossible to unequivocally identify single causative variants because many variants may share the same SDP. Bearing in mind these caveats, we assessed the correlation between sequence and DHS strain distribution patterns at all variable sites. 503/1397 (36%) of DHS are associated with a sequence variant located within the site or 100 bp either side at an FDR of 5%. 165 of the variable sites (12% of the total) contained one or more variants that either disrupted a known motif for transcription factor binding site or created a novel one in one or more strains.

We chose to search for variants within 100 bp of the DHS to take into account the fact that nucleosome occupancy at the edge of the sites is not entirely fixed. Ideally we would like to look further, but our ability to do so is limited by the small number of haplotypes we are examining in the eight inbred strains (there are only 127 possible combinations of di-allelic variants in 8 strains). The further we extend our search out from the DHS, the greater the chance that we will find an association by chance. Figure S2 shows a linear increase in the percentage of associated sequence variants (up to to 70% at 100 Kb). We are unable to tell which of these represent true associations.

We asked next whether sequence variants potentially causal for site variation are more likely to be found for discretely varying sites (the set of 196 sites where there is either total loss or gain of a site in at least one strain). Of the 196 DHS, 113 (57%) contain a sequence variant within 100 bp of the site whose strain distribution pattern correlates with that of the variable DHS (at an FDR of 5%). This is significantly different from the set of continuously varying (P-value 1.18e-8, Fisher exact test). Four discretely varying DHS coincide with a structural variant (one deletion and three insertions) and 42 (21%) contain a variant in a known transcription factor binding site.

What are the functional consequences of alteration in DHS? To evaluate this we analysed the relationship between DHS variation and expression of RNA in erythroblasts. Using RNA-Seq, genome-wide RNA abundance and inter-strain variability was estimated in erythroblasts from the same eight strains used for the DHS analysis. We hypothesised that changes in both RNA expression as well as RNA processing are more likely to be found associated with variable DHS than with non-variable DHS.

**Figure 3:**
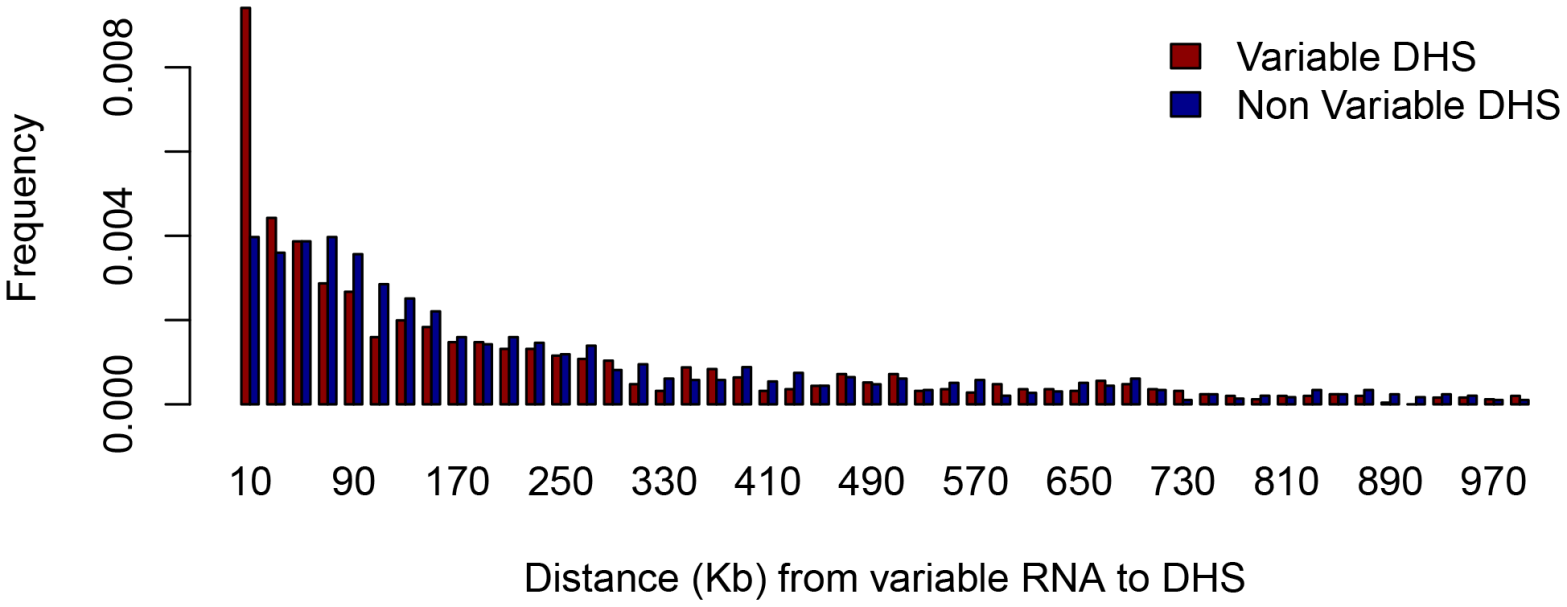
Proximity of DNase I hypersensitive sites to variable RNA. The histogram shows the relative abundance of DHS within one megabase (Mb) of the transcriptional start site of variable RNA species. The horizontal scale is in kilobases (Kb). Variable RNA refers to the 3,472 poly(A)^+^ transcripts that varied between strains (FDR of 5%). Variable DHS refer to the 1,396 variable peaks (as described in the text), and non-variable refers to 2,849 with least evidence for variation.

We identified 3,472 poly(A)^+^ transcripts that varied between strains (using an FDR of 5%). We calculated the shortest distance from the start of each variable transcript to variable DHS and to non-variable DHS (the 2,849 least variable, P-value <0.008). Within 10 Kb of any variable poly(A)^+^ transcript there are a total of 181 variable DHS (13% of the total) compared to 85 non-variable (P-value = 3.4e-91, Fisher exact test) (Figure 3). Proximity of variable RNA and variable DHS does not mean the two events are correlated so we tested whether this was so. We found that quantitative variation in peak height at 54 of these 181 variable DHS lying within 10 Kb of variable poly(A)^+^ RNA transcripts was significantly (P-value <0.05, uncorrected) correlated with variation in RNA abundance. If we assume that the effect of a variable DHS on a transcript is limited to transcripts within 10 Kb, then this result indicates that at most 4% (54) of the 1,396 variable DHS affect transcription. This is a conservative estimate since the analysis does not take into account the number of false discoveries (5% of 181 = 36).

Our findings appear to indicate that over 95% of the variable DHS are not related to readily detectable variation in gene expression. We investigated this further in three ways. We analysed the set of DHS for which an effect on transcription is likely most easily observed, namely that set of 196 sites with either total loss or gain of a site in at least one strain (this is the set of DHS with discrete, rather than continuous variation). Second, we examined the effect of discrete DHS variation over a larger interval, up to 300 Kb from the site. Finally we extended our analysis to the poly(A)^−^ fraction for the set of discretely varying DHS. Poly(A)^−^ RNA represents nascent transcripts both from genes and regulatory elements. Its analysis provides a more dynamic and comprehensive assessment of RNA expression.

**Figure 4:**
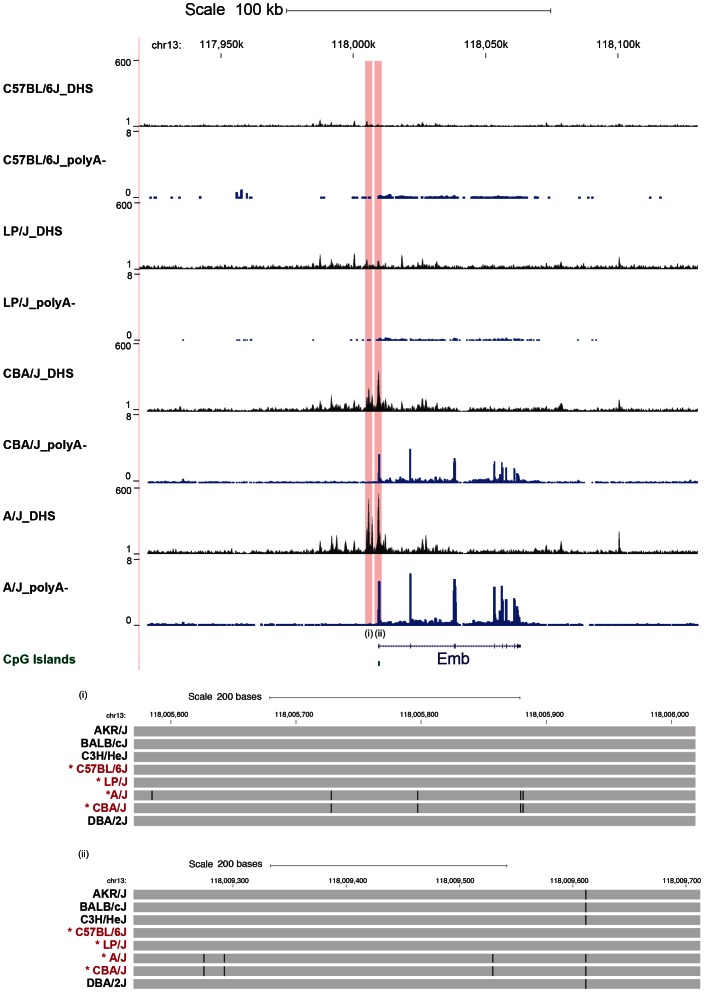
Relationship between variable DNase I hypersensitive sites, underlying sequence and transcript variation. Scale and position in the MGSC37 mouse reference are shown as before. The density of aligned DNase-seq reads in a moving 300 bp window, with a 30 bp increment, is shown for the mouse strains C57BL/6J, LP/J, A/J and CBA/J. The poly(A)^−^ transcription from each strain is displayed (in blue) as the aligned read depth per base pair normalized per million of aligned reads. The two variable DHS (one at the *Emb* gene promoter and one upstream of the promoter) are highlighted in red. The strain specific genotype of the DNA underlying the variable peaks is shown below (regions indicated by roman numerals) with a grey bar representing the portions identical to the current mouse genome build; differences are represented as black lines.

We visually inspected 300 Kb either side of the 196 discretely varying peaks for poly(A)^+^ and poly(A)^−^ transcriptional changes. An example is shown in Figure S3. At 14 loci (7%) the gain or loss of a DHS was associated with a change in gene expression. For example, at the *Emb* gene (embigin, a transmembrane protein of the immunoglobulin super-family class of cell adhesion molecules) the occurrence of two DHS, one at the transcriptional start site and another 3,600 bp upstream, are associated with transcription of the gene (Figure 4). Thus approximately 7% of variable DHS of the peaks with discrete variation are associated with changes in transcription (either poly(A)^+^ or poly(A)^−^). The DNA sequence variants associated with the presence or absence of DHS peaks are clearly correlated with the presence or absence of RNA transcripts (Figure 4).

**Figure 5:**
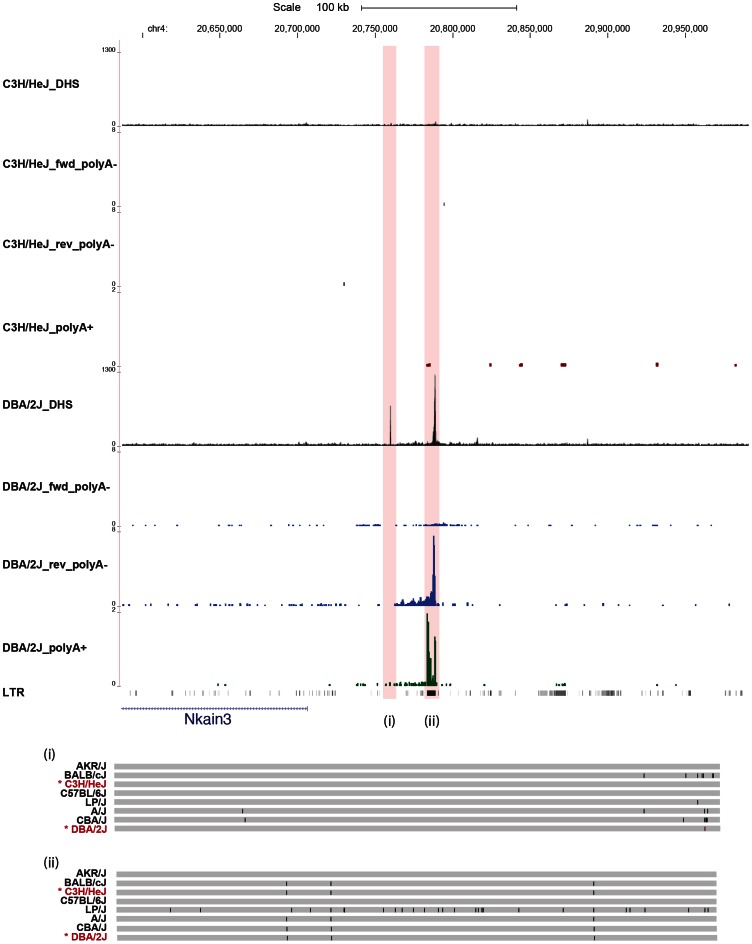
Some variable DNase I hypersensitive sites have no underlying sequence variation. The figure shows one example on chromosome 4 where a DHS, present only in strain DBA/2J, is associated with both poly(A)^−^ (blue) and poly(A)^+^ (green) transcription, in the absence of sequence variation consistent with the strain distribution of the DHS. The chromosomal position in is shown in Kb. The density of aligned DHS-seq reads in a moving 300 bp window, with a 30 bp increment is shown for strains C3H/HeJ and DBA/2J. The strain specific genotype of the DNA underlying the variable peaks is shown below with a grey bar representing the portions identical to the current mm9 build; differences are represented as black lines. Note that there are no differences between C3H/HeJ and DBA/2J.

Our results do not exclude the possibility that some DHS alter transcription without the presence of a sequence variant. We searched for examples by re-examining the entire set of 36,693 DHS. We identified 42 additional discretely varying peaks that did not appear to contain correlated sequence variants. In one case, we found a DHS present only in one strain (DBA/2J) associated with both poly(A)^+^ and poly(A)^−^ transcription (Figure 5). The novel transcript contains a re-activated LTR repeat element. No variants were detected consistent with the strain distribution pattern of the DHS (Figure 5) (failure to find variants could not be attributed to low sequence coverage or read mapping errors). Thus, in some circumstances, changes in both DHS and expression can occur in the absence of local sequence variation.

We then investigated the relationship between DHS and phenotypic variation. To do so, we took advantage of a prior analysis in which variants in the genomes of the eight strains analysed here had been tested for causal involvement at each of 843 quantitative trait loci (QTLs) for over 100 different phenotypes [12] [13]. The results provide data similar to that from human genome wide association studies, namely a set of positions in the genome that are candidate functional variants.

We asked if variable DHS that coincide with QTLs are enriched for candidate variants. Within the 1,397 variable DHS we identified 199 candidate variants and 2,614 non-candidate variants. Within the 2,849 non-variable DHS we identified 188 candidate variants and 3,220 non-candidate variants. This difference is significant P-value = 0.013 (Fisher's exact test). Restricting the analysis to variants at QTLs relevant to haematological phenotypes (mean cellular haemoglobin, mean cellular volume, red blood cell count and haemoglobin concentration) we again observed an enrichment (3 candidate variants in the variable DHS compared to none in the non-variable DHS) with a P-value of 0.08 (Fisher's exact test).

## Discussion

We have shown that among eight mouse inbred strains about 9% of DHS are variable. The majority of these variable sites cannot be explained by a sequence variant either within the site, or 100 bp either side, thus ruling out simple models in which a sequence variant locally alters chromatin conformation. In our assays, the functional consequences of site variation are relatively limited: only 7% are correlated with changes in transcriptional activity. Finally we find that sequence variants within variable DHS are 30% more likely to be associated with complex traits than sequence variants lying in non-variant DHS. We discuss these points below.

Our estimate of the proportion of DHS that is variable is consistent with those obtained from human lymphoblastoid cell lines [5], [6]. However we have reported fewer sites than identified in ENCODE cell lines (ranging from 84,201 to 266,618 total sites per cell line) [3]. This may be because there are fewer DHS in primary differentiated cells than in immortalised cell lines; and that we chose to concentrate our analysis on high confidence peaks, reducing the chances that our detection of variable peaks would include false positive peaks.

About 65% of variable sites are not correlated with sequence variation. Even when we limit our observations to instances where a site is lost or gained, at a third of peaks we are still unable to attribute this alteration to a variant lying under the peak or within 100 bp. One possibility is that sequence variation within distal regulatory elements result in changes in interactions with the variant DHS. Such interactions, in which widely separated regions of the genome come into contact with each other via regulatory elements, would explain the occurrence of clustered sites (e.g. Figure 2b). Trans-acting effects and heritable epigenetic changes (such as DNA methylation) may also contribute. Our study, like others, is not sufficiently powered to detect this source of genetic variation [8].

We identified a potential molecular mechanism for DHS variation (the disruption of a TFB site or a structural variant coinciding with a DHS) at 165 sites (12% of the total). While this analysis cannot unequivocally identify which variants are causal, it is consistent with recently published ENCODE results indicating the importance of sequence variation at a proportion of DHS [2], [3]. However in one case we observed the presence of a DHS in a single strain, associated with a novel transcript, without an underlying sequence change, indicating that sequence variation at the DHS alone is an insufficient explanation both for DHS change and its functional consequences.

Indeed the functional consequences of DHS variation from our data appear relatively limited. We found that less than 10% of variable DHS are likely to have an effect on transcription. However we cannot exclude the possibility that the effect of DHS variation is broader than we have observed, having an effect only in different environments or tissues to those assayed, or that the effects are too subtle to have been detected.

It is possible that the relative absence of causative sequence variants and the difficulties finding functional correlates of site variation might arise in part from the fact that site variation reflects some unknown but consistent measurement artefact, or is simply a biologically unimportant consequence of chromatin structure around DHS. The bulk of DHS variation we observe is continuous, so that we resort to using a probability cut-off to determine what is variable and what is not.

To counter this problem, we analysed separately the 196 sites with discrete, rather than continuous variation. These DHS show total loss or gain of a site in at least one strain. Our analyses of the discrete sites support results from the entire set of variable sites, namely only a small fraction (approximately 7%) are associated with changes in transcription (either poly(A)^+^ or poly(A)^−^). A third of 196 DHS lack sequence variants that can explain strain distribution pattern correlates with that of the variable DHS.

Finally, our data address the question whether sequence variants underlying variable DHS contribute to complex phenotypes. Functional sequence variants are indeed enriched in DHS. We have shown that sequence variants at variable DHS are more likely to contribute to phenotypic variation than sequence variants within non-variable DHS, consistent with the finding that DHS are enriched with signals from human genome wide association studies [9]. Our data suggest that searching for causal variants of complex traits will profit by focusing within variable DHS, but such an approach will have to survey many tissues, presumably at different developmental time points and under a variety of environmental circumstances, to find a substantial fraction of the sequence variants involved in complex traits.

## Materials and Methods

### Primary cells

Mature primary erythroid cells were obtained from phenylhydrazine-treated adult (6–9 weeks old) female mice as described [19]. Spleens containing erythroblasts were mechanically disrupted to single cell suspension and erythroblast purity was between 60–80%. Mice for this experiment were the progenitors of the heterogeneous stock (HS) (A/J [JAX #646], AKR/J [JAX #648], BALB/cJ [JAX #651], CBA/J [JAX #656], C3H/HeJ [JAX #659], DBA/2J [JAX #671] and LP/J [JAX #676]).

### DNase-seq

DNase I assays were performed using ∼5×10^7^ cells. Nuclei from mainly terminally differentiated erythroblast (∼60–80% cells) were digested with eight different concentrations of DNase I (0_ice_, 0_37_, 0.5, 1, 2, 4, 8, 16, 32, 64 µl of 10 U/µl DNase I in 1 ml reaction volume) at 37°C (except 0_ice_) until the sample containing 16 µl reached water-like viscosity. The digestions were stopped instantly by transferring the tubes to an ice box followed by transferring the reactions into lysis buffer. DNA was extracted using a phenol/chloroform mix and ethanol precipitated. Since DHS represent a spectrum of signal intensities, and the optimal sample should include material from different DNase I concentration digestions, the mid phase of digestions were selected for library preparation by qPCR (see below). Based on the principle that DNase I may cut more than once across DHS, generating small DNA fragments that can be amplified, Solexa compatible libraries were constructed as follows: DNA (2–4 µg) was blunt-ended with T4 DNA Polymerase (NEB), adenine overhangs were added using Klenow 5′-3′ exo-minus, Illumina Solexa sequencing adapters were ligated using T4 DNA ligase (NEB) and amplification was carried with 10 PCR cycles (15 sec extension time) using Phusion DNA polymerase (Finnzymes) and Illumina Solexa sequencing primers 1.1 and 2.1 (Illumina). Finally, libraries were size selected by electrophoresis and sequenced on an Illumina Genome Analyzer (GA-IIx) or HiSeq2000.

### Quantification of DNase I digestion by qPCR

To assess DNase I digestion in terminally differentiated erythroblasts, three pairs of forward and reverse primers were designed to amplify ∼140–160 bp products in murine positive (MCS-R2, MCS-R3, minus14) and negative (Control-1, Control-2, Control-3) DHS. The efficiency of the primers, their uniqueness and whether or not they contain any SNP in different 8 strains were interrogated. MCS-R2 and MCS-R3 are two of the regulatory elements of the α-globin genes [20]. Minus14 amplifies the promoter of a housekeeping gene (Nprl3) within the murine α-globin regulatory domain. Controls one and two are regions in the α-globin locus which appeared inactive using H3K4me2 ChIP on chip. Control-3 measures the *Pkdrej* gene, which is sperm specific and is very condensed in all tissues. Among these, the best positive and negative set of primers were chosen for qPCR based on the similar efficiency of test and control amplification, and the specificity of primers from melting curve analysis and visualizing the PCR products on agarose gels. The standard curves were used to calculate the percentage of copies of the MCS-R3 amplicon remaining in 5 ng of DNase I-treated genomic DNA in different phases of digestion (different DNase I concentrations).

The amount of template DNA was standardized by correcting for amplification of the DNase I-insensitive Control-2 or Control-3 sequence [21]. From three replicates of DNase I digestion per strain, mid-phase of digestion of the sample was selected for GAII library preparation. PCR was performed using SYBR green PCR iQTM SYBR Green Supermix (BioRad), and real-time PCR was performed on a iCycler iQTM real-time PCR detection system (BioRad). Genomic DNA was quantified three-times by using the NanoDrop 2000 Spectrophotometer (Thermoscientific) and a PicoGreen dsDNA Assay kit (Invitrogen). Five nanograms of genomic DNA was digested with eight different concentrations of DNase I and loaded onto 96-well optical PCR plates by using a iCycler machine. PCRs performed in triplicate generated highly reproducible results (SD≤0.2); outliers (>3SD) were excluded from the analysis. A similar approach was applied to design qPCR in C57BL/6J mouse embryonic fibroblasts (MEFs). To improve the efficiency of the primers, ∼110–120 bp primer sets were designed to capture regulatory sites at 2 housekeeping genes (*Gapdh* and β-actin) as positive DHSs in MEFs and 3 negative sites (Control-1–Control-3 as previously described).

### Sequence alignment

In order to mitigate the impact of inter-strain sequence variation on mapping quality we first created pseudo genomes for each of the eight strains by introducing single nucleotide polymorphisms from the relevant strain into the MGSC37 mouse reference [10]. We then aligned reads from each lane to the appropriate mouse strain genome using STAMPY [22]. Purpose-written software was used to calculate the genomic coverage for DNA fragments represented by each read pair. In particular, for short fragments where the two reads of a pair overlap, sequencing primers were removed and bases of the overlapping region were only counted once. Conversely, the coverage included the entire aligned DNA insert and not merely two ends sequenced in the read pair. Read map alignments were filtered for quality, removing reads with a mapping quality values <2. Alignments to repetitive parts of the genome were filtered by excluding genomic regions with a CRG 50-mer Alignability score of <0.5 [23]. These are duplicate regions that are present in more than two (near) identical copies in the genome.

### Peak detection and analysis

We called DNase I hypersensitive sites using purpose-written software. Let *X_j_* be the count of aligned reads covering position *j* in the genome. To allow for local variation in the coverage of aligned reads, we computed the deviation *d_j_* of the overall mean coverage from the local mean coverage (in a window of width 2*L*+1) and adjusted *X_j_* to *W_j_* = *X_j_*−*d_j_*+μ where μ is the overall mean coverage. This maintains the same average read coverage, μ. A small number of sites where the adjusted values were negative were truncated to 0. Then we called peaks on the transformed scores *Y_j_* = log*_2_*(*W_j_/pμ*) where the parameter *p* controls the stringency of the peak detection. We called peaks by searching for high-scoring segments [16], defined as intervals [*m,n*] such that *S_mn_* = Σ_m<j<n_ *W_j_*, satisfies the conditions *S_mn_*>0 and *S_mn_*>*S_uv_* where [*u,v*] is any interval containing [*m,n*]. *W_j_* is negative whenever *X_j_-d_j_<pμ*, so we only call peaks in regions where adjusted counts are at least *p* times the mean coverage. The motivation for the definition of Y is first that Y is effectively independent of the average coverage (so that we can compare data with different read coverage, e.g. from an Illumina GA-II and a HiSeq2000) and second that peaks with extremely high values (e.g. to due to unresolved repeats being mapped to the same locus) carry relatively little additional weight.

Variation between peaks was estimated under the assumption that the distribution of reads at a peak can be modeled as a negative binomial distribution. We used the DESeq package to estimate the differences between peaks in different strains [17]. Correlation between peaks and sequence variation was performed in linear models using the statistical software package R. The strain distribution pattern of a variant was expressed as a vector, so that for example a sequence variant present only in strains A/J and LP/J would be the vector ABBBBBBA (where strains are in the order A/J, AKR/J, BALB/cJ, C3H/HeJ, C57BL/6J, CBA/J, DBA/2J, LP/J). We asked if the same vector is present in the pattern of strain variation of DHS peaks.

The degree of variation was estimated by the heritability of the site, defined as half the between strain component of variance divided by the sum of the within strains variance and half the between strain variance [18]. Although the distribution of peak heights violates some assumptions behind this calculation, the heritability provides an intuitive measure of the extent of variation.

Peak coordinates were associated with the following genomic features: sequence variants identified in the eight inbred progenitor strains of the HS [10], [11]; vertebrate sequence alignments taken from the multiz30way track of the UCSC genome browser [24]; regions enriched for H3K4me1 (enhancer feature) and H3K4me3 enrichment (promoter feature) [15]; transcription factor binding motifs from TRANSFAC database identified by running FIMO [25] with a detection threshold of P-value <10^−5^ (FIMO was run on the MGSC37 mouse reference and on sequences for each strain by introducing single nucleotide polymorphisms into the reference sequence (as described above). Statistical analyses were carried out using the R software package [26].

### RNAseq

For RNA-Seq libraries, total RNA was split into poly(A)^+^ and poly(A)^−^ RNA using the PolyATract mRNA isolation system (Promega). Poly(A)^+^ RNA libraries were generated using the Illumina mRNA-Seq paired-end kit after globin depletion using GlobinClear (Ambion). Poly(A)^−^ RNA libraries were generated using the ScriptSeq v2 RNA-Seq Library Preparation Kit (Epicentre), after depletion of ribosomal transcripts with RiboMinus Eukaryote Kit for RNA sequencing (Invitrogen). Poly(A)^+^ and poly(A)^−^ libraries were sequenced on Illumina HiSeq2000 and GA-IIx respectively. Read alignments were filtered for quality using samtools version 0.1.18 [27] and reads with a MAPQ value <15 were removed. Sequence reads that overlap with transcript intervals were counted using the R package IRanges [26] and a table of transcript and exon coordinates provided by UCSC, based on the mouse reference genome assembly mm9. Reads were mapped to n = 26347 (out of 37681) transcripts. Normalization of read numbers was carried out using DESeq version 1.8.3 [17]. Transfrags were assembled de-novo using Cufflinks version 1.3.0 [28] (n = 74390 transfrags) and these transfrags were linked to the reference genome using cuffcompare. Differential expression of transcripts in the strains used was analysed using cuffdiff.

### Ethics statement

All animal work was conducted according to UK guidelines and approved by the UK Home Office.

## Supporting Information

Figure S1 Effect of altering parameters in the peak finding algorithm on the detection of variable peaks. The two parameters are the window size (in kilobases), shown on the horizontal axis, and the stringency (p), shown for values between 2 and 8 as a set of coloured lines. The vertical axis shows the percentage of variable sites, as calculated using a 5% FDR threshold. The number of variable peaks at each combination of parameter settings was estimated using the DESeq package as described in the Materials and Methods section of this paper.
(PDF)

Figure S2 Closest distance from a variable DHS to a significantly associated sequence variant. The horizontal axis shows the distance in kilobases (Kb) from each of the 1,397 variable DHS to a sequence variant that is associated at a 5% FDR. The P-value is calculated by associating the strain distribution pattern of the DHS with the strain distribution sequence variant. We report here the distance to the closest variant (because of the haplotype structure of the inbred mouse genome there are often large haplotype blocks with identical strain distribution patterns). The vertical axis shows the percentage of DHS for which an association is found.
(PDF)

Figure S3 Relationship between variation in transcription and a variable cluster of DHS peaks. Chromosomal positions are shown in kilobases. The density of aligned DHS-seq reads in a moving 300 bp window, with a 30 bp increment is shown for each mouse strain. The poly(A)^−^ transcription from each strain is displayed (in blue) as the aligned read depth per base pair normalized per million of aligned reads.
(PDF)

Table S1 Table of variable DHS and associated features. The 1,397 peaks are categorized into three classes: I discrete simple; II discrete compound; III continuous quantitative variation. The table provides results from analysis of matches to regions of sequence conservation, to regions identified as enhancers and promoters (is.enh and is.promoter), and the names of genes closest to the peak (ENSEMBL id and MGI ID). The last three columns provide information about the degree of variation between strains. The first column is the P-value for the analysis that models the distribution as a negative binomial (from the DESeq package). The next column is the heritability and the last the P-value of the heritability.
(XLSX)
