# Supplementary figures and images for: Causes and Consequences of Chromatin Variation between Inbred Mice
Source: PLoS Genet. 2013 Jun 13;9(6):e1003570. doi: 10.1371/journal.pgen.1003570 (PMC3681629; doi:10.1371/journal.pgen.1003570)

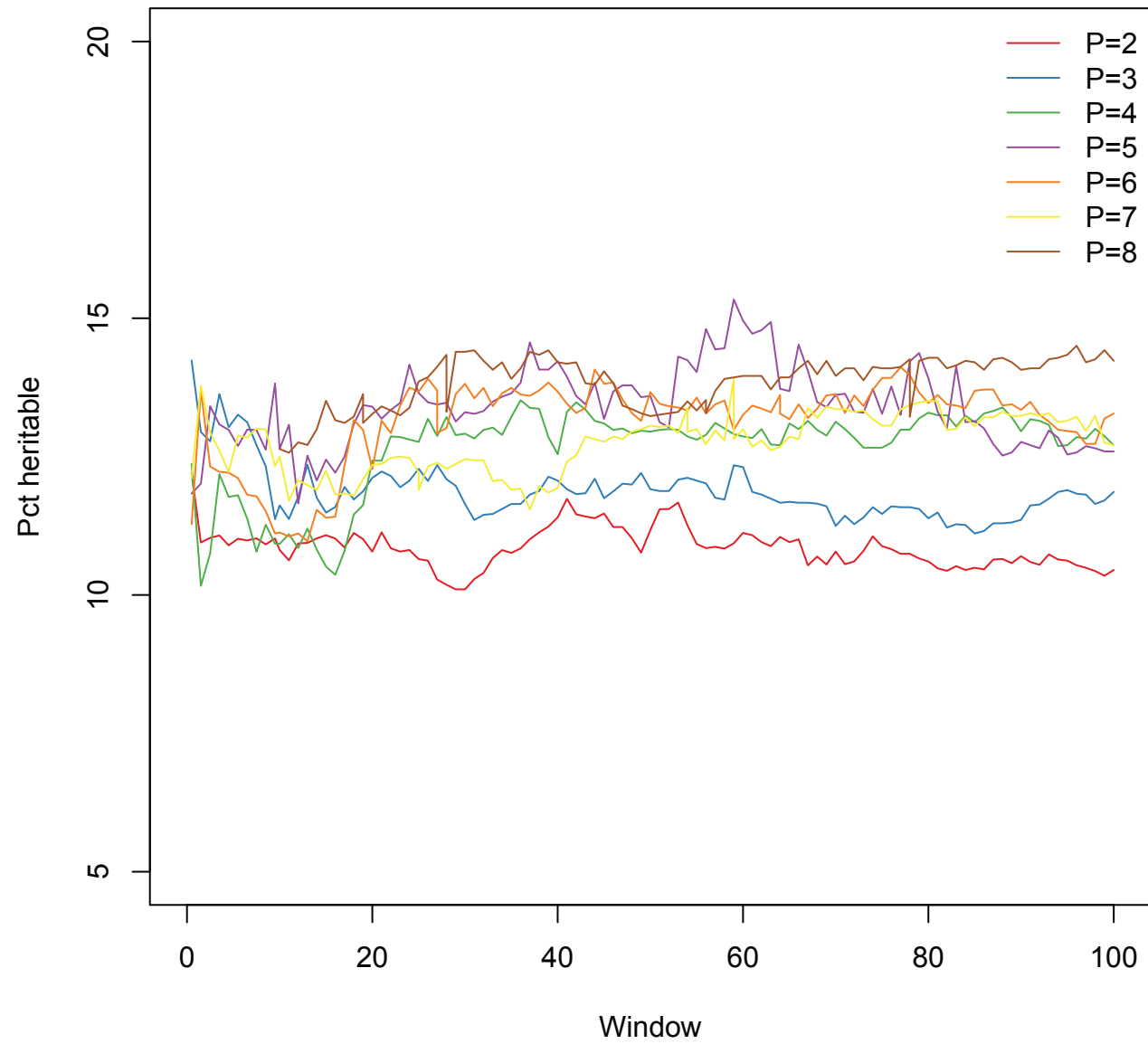

Supplementary Figure 1

Supplement: Figure S1 — Effect of altering parameters in the peak finding algorithm on the detection of variable peaks. The two parameters are the window size (in kilobases), shown on the horizontal axis, and the stringency (p), shown for values between 2 and 8 as a set of coloured lines. The vertical axis shows the percentage of variable sites, as calculated using a 5% FDR threshold. The number of variable peaks at each combination of parameter settings was estimated using the DESeq package as described in the Materials and Methods section of this paper. (PDF) [file pgen.1003570.s001.pdf]

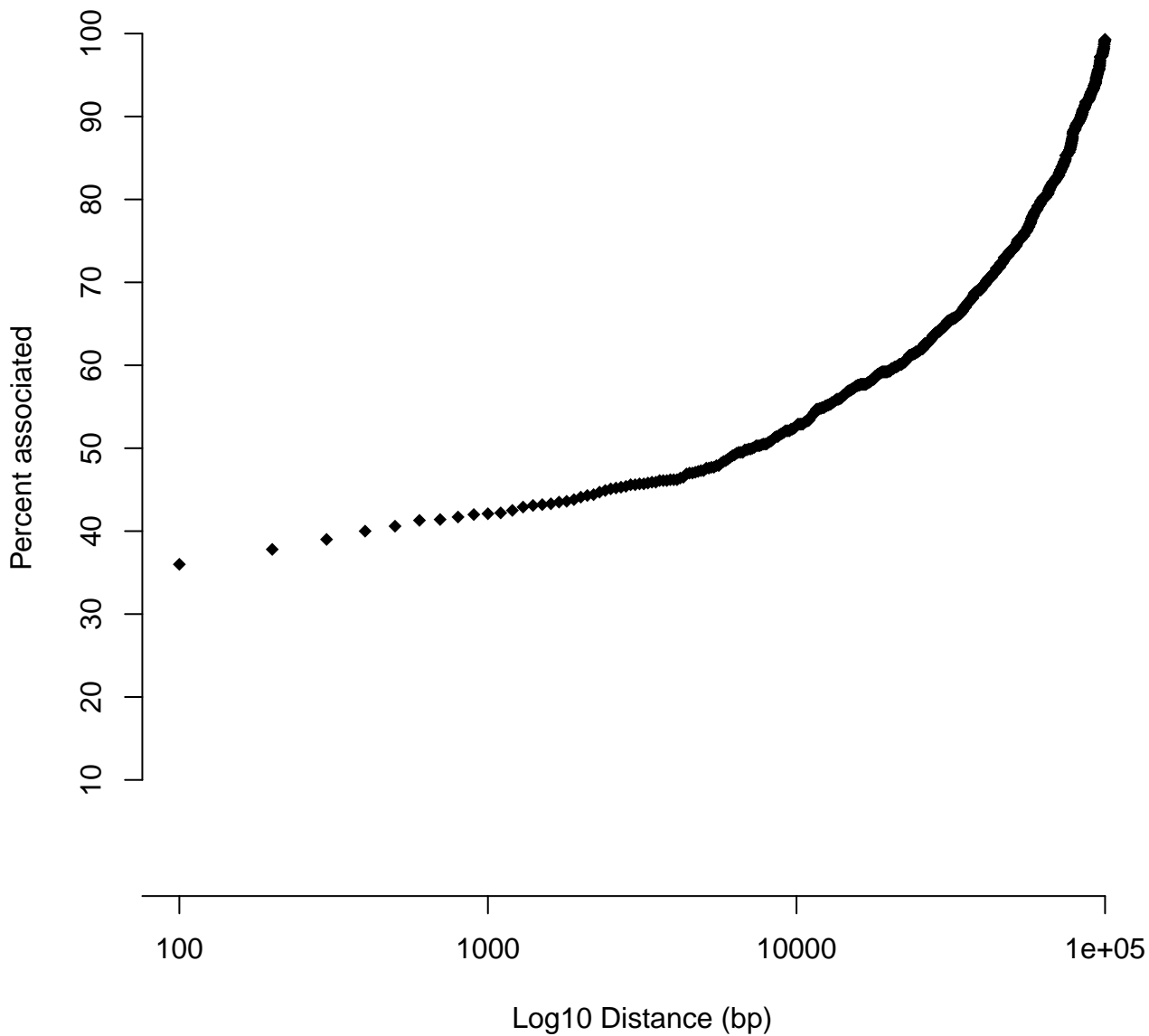

Supplement: Figure S2 — Closest distance from a variable DHS to a significantly associated sequence variant. The horizontal axis shows the distance in kilobases (Kb) from each of the 1,397 variable DHS to a sequence variant that is associated at a 5% FDR. The P-value is calculated by associating the strain distribution pattern of the DHS with the strain distribution sequence variant. We report here the distance to the closest variant (because of the haplotype structure of the inbred mouse genome there are often large haplotype blocks with identical strain distribution patterns). The vertical axis shows the percentage of DHS for which an association is found. (PDF) [file pgen.1003570.s002.pdf]

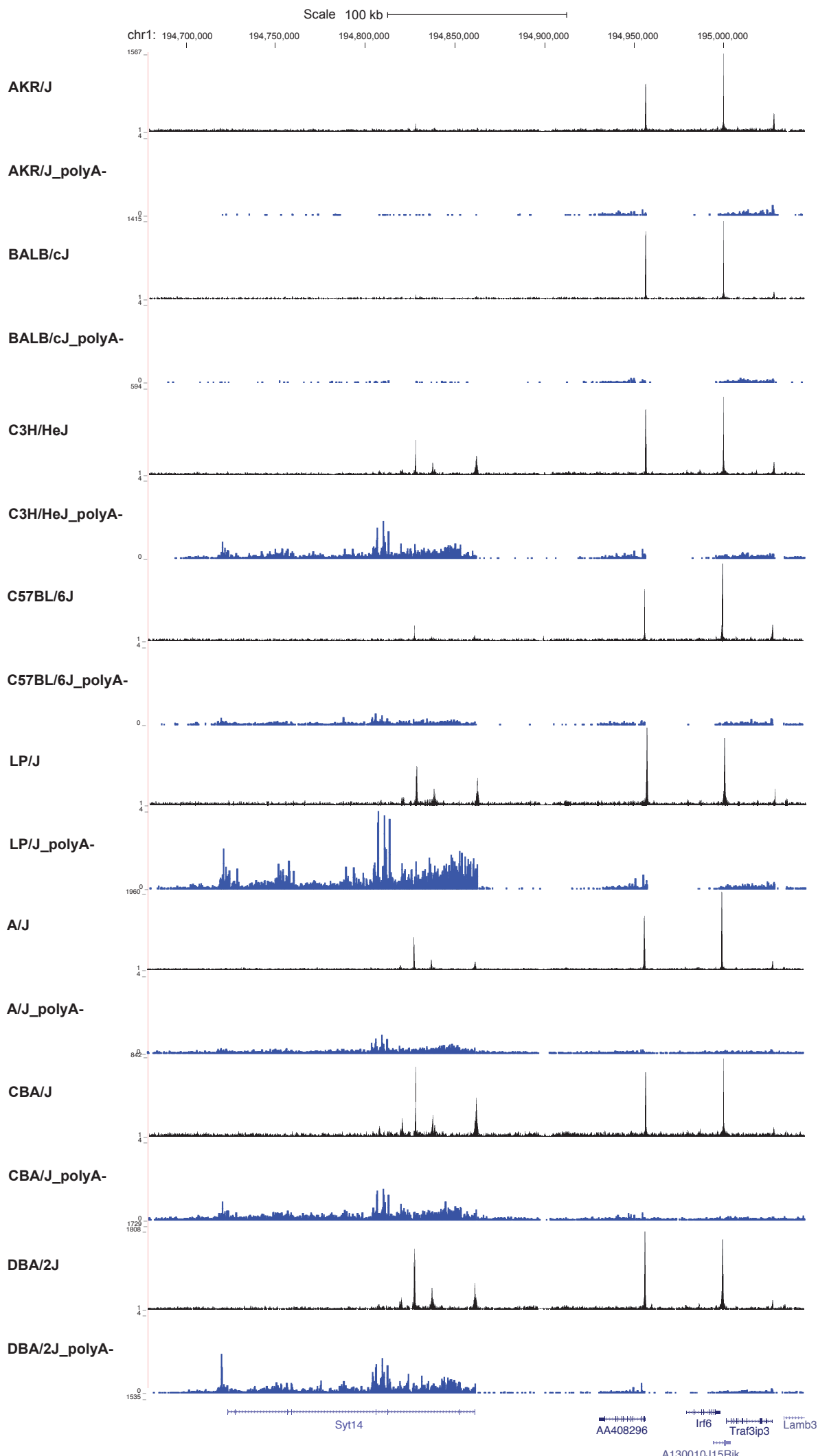

Supplement: Figure S3 — Relationship between variation in transcription and a variable cluster of DHS peaks. Chromosomal positions are shown in kilobases. The density of aligned DHS-seq reads in a moving 300 bp window, with a 30 bp increment is shown for each mouse strain. The poly(A)− transcription from each strain is displayed (in blue) as the aligned read depth per base pair normalized per million of aligned reads. (PDF) [file pgen.1003570.s003.pdf]
